# Polyphenol Profile and In Vitro Antioxidant and Enzyme Inhibitory Activities of Different Solvent Extracts of Highland Barley Bran

**DOI:** 10.3390/molecules28041665

**Published:** 2023-02-09

**Authors:** Wengang Zhang, Yongli Lan, Bin Dang, Jie Zhang, Wancai Zheng, Yan Du, Xijuan Yang, Zhonghong Li

**Affiliations:** 1 College of Food Science and Engineering, Northwest A&F University, Yangling 712100, China; 2 Laboratory for Research and Utilization of Qinghai Tibet Plateau Germplasm Resources, Qinghai Tibetan Plateau Key Laboratory of Agricultural Product Processing, Qinghai Academy of Agriculture and Forestry Sciences, Xining 810016, China; 3 Academy of Agriculture and Forestry Sciences, Qinghai University, Xining 810016, China; 4 Qinghai Province Highland Barley Resources Comprehensive Utilization Engineering Technology Research Center, Qinghai Huashi Science & Technology Investment Management Co., Ltd., Xining 810016, China

**Keywords:** highland barley bran, phenolic compounds, antioxidant activity, α-glucosidase, α-amylase

## Abstract

Five different solvent extracts of highland barley bran were analyzed and compared for their polyphenol profile, antioxidant activity, and α-glucosidase and α-amylase inhibitory activities. The highland barley bran acetone extract had the highest total phenolic content, total flavonoid content, and antioxidant capacity. It was followed by the methanol and ethanol extracts, while n-butanol and ethyl acetate extracts exhibited lower measured values. Diosmetin, luteolin, protocatechuic acid, vanillic acid, ferulic acid, phlorogucinol, diosmin, isoquercitrin, catechin, and isovitexin were among the most abundant phenolic compounds identified in different solvent extracts, and their concentrations varied according to the solvent used. The highest α-glucosidase and α-amylase inhibitory activity were observed in the ethyl acetate extract of highland barley bran, followed by the acetone and methanol extracts. In contrast, n-butanol and ethanol extracts exhibited lower measured values. The different solvent extracts were effective inhibitors for α-glucosidase and α-amylase with activity reaching to 34.45–94.32% and 22.08–35.92% of that of positive control acarbose, respectively. There were obvious correlations between the phenolic content and composition of different solvent extracts and their in vitro antioxidant activity, α-glucosidase inhibition activity and α-amylase inhibition activity. Black barley bran is an excellent natural raw material for developing polyphenol-rich functional foods and shows good antioxidant and hypoglycemic potential to benefit human health.

## 1. Introduction

Highland barley (*Hordeum vulgare* L. *var. nudum* Hook. f.) is a cereal crop of the *Hordeum* genus that has a long cultivation history in China and is primarily cultivated in high-altitude areas (4200–4500 m) in Tibet, Qinghai, Yunnan, Sichuan, and Gansu [1,2]. Highland barley is rich in nutritional and functional compounds, with the characteristic nutritional profile of “three highs and two lows” (high fiber, high protein, high vitamins, low fat, and low sugar), consistent with modern healthy dietary requirements [3]. Highland barley germplasm resources are abundant and are classified as black, yellow, blue, purple, etc., according to grain color. Each colored grain type has a unique nutritional and functional composition [4]. Colored highland barley, particularly black highland barley, is rich in important functional components such as polyphenols, anthocyanins, and β-glucans. It also appears to have a higher overall edible value than common highland barley, thus receiving increasing attention from the food processing industry [5]. Regular consumption of whole-grain highland barley improves glucose and lipid metabolism disorders in humans and can contribute to the prevention of obesity and cardiovascular disease [6]. After milling, highland barley is primarily used as a functional raw material and ingredient in various food products. However, the milling rate of highland barley is low, and a large amount of bran is sloughed down during the milling process, mostly used as a feed or discarded, resulting in significant resource waste [7].

The major challenges confronting modern society are hyperlipidemia, hyperglycemia, cardiovascular disease, inflammation, and cancer. Their development and occurrence are thought to be closely linked to oxidative stress [8]. Furthermore, certain enzymes, such as α-amylase, α-glucosidase, lipase, angiotensin-converting enzyme, urokinase, and pancreatic enzymes, are considered promising drug targets for metabolic diseases prevention and treatment [9,10,11]. Polyphenols, a class of aromatic hydroxy derivatives, have strong antioxidant activity and can inhibit free radical-induced polymerization chain reactions. They are natural, efficient, safe, low-cost antioxidants and enzyme inhibitors compared to synthetic antioxidants, ideal for naturally preventing and improving these diseases [12,13]. Polyphenol compounds (such as flavonoids, anthocyanins, and phenolic acids, among others) in highland barley are primarily concentrated in the bran portion, providing exceptional biological activity and the potential to be used as an excellent dietary polyphenol supplement, with increasing interest in functional food development [14,15,16]. In general, plant polyphenols must be extracted and enriched using organic solvents with varying polarities. Methanol, ethanol, acetone, ethyl acetate, petroleum ether, n-butanol, and other commonly used solvents are used for extraction. There are differences in the polyphenol content, composition, and biological activity in extracts of different solvents [10,17,18]. Lee et al. investigated the anthocyanin in vitro activity from four different colored barley grains (purple, blue, black, and yellow). They discovered that purple-grain barley bran extract exhibited the highest DPPH radical, superoxide radical scavenging ability, and overall antioxidant activity, with a lower IC_50_ concentration on angiotensin I converting enzyme (ACE) than purple barley grain extract [19]. Abuarab et al. prepared barley bran ethanol, n-hexane, water–methanol, and water extracts. They discovered that the n-hexane and water–methanol extracts resulted in significant regulatory immunocompetence in mouse experiments, whereas the ethanol and n-hexane extracts exhibited significant anticancer efficacy [20]. Moreover, hot water, cold water, and 12% ethanol extracts from 13 different varieties of barley exhibited a significant in vitro inhibitory activity against α-amylase and α-glucosidase, with black barley showing the highest α-glucosidase inhibitory activity at 34%. According to this study, the barley polyphenol extract may be beneficial in combating hyperglycemia and oxidative stress associated with early type 2 diabetes (T2D) [21]. As a result, it is evident that the phenol-rich barley (highland barley) bran has a high potential for promoting overall health and wellbeing [22,23].

A number of studies have evaluated the in vitro and in vivo activities of different solvent extracts of highland barley bran. However, the differences of phenolic composition, antioxidant activity, α-glucosidase inhibitory activity, and α-amylase inhibitory activity of different extracts are not clear. In addition, the relationship between the phenolic compounds recovered in the extracts and their activities remains unknown, limiting the application and utilization of highland barley bran in healthy foods and supplements. To this end, the purpose of our study was to analyze and compare five different solvent extracts of highland barley bran in terms of their phenolic content and composition, in vitro antioxidant capacity, α-glucosidase inhibitory activity, and α-amylase inhibitory activity. The relationship between polyphenol compounds and antioxidant capacity and enzyme inhibitory activity of different extracts was comprehensively assessed. This research contributes to the theoretical foundations for the scientific development and utilization of highland barley bran in human nutrition and the value of highland barley resources.

## 2. Results and Discussion

### 2.1. Phenolic Content Analysis

The total phenolic contents of the different highland barley bran solvent extracts ranged from 197.93–281.98 mg/100 g DW. The acetone group showed the highest total phenolic content, and no significant differences were observed between the ethanol and methanol groups (*p* > 0.05) and between the n-butanol and ethyl acetate groups (*p* > 0.05). (Table 1). Liu et al. prepared 60% acetone extract, 80% methanol extract and water extracts of highland barley bran and found that the 60% acetone extract had the highest total phenolic content (407.52 mg/100 g), followed by 80% methanol extract (192.10 mg/100 g) and water extract (190.21 mg/100 g), which was similar to the phenomenon found in this paper [24]. The total phenolic content reported by Liu et al. in 60% acetone extract of highland barley bran was higher than in this work, and the main reason might be that there were differences in raw material, solvent concentration and extraction method. The total flavonoid content in the different barley bran solvent extracts ranged from 2.52 to 12.22 mg/100 g DW, with the acetone group exhibiting the highest value and no significant differences observed between the ethanol and methanol groups (*p* > 0.05). The total phenolic and flavonoid content of the different solvent extracts was in the order of acetone > ethanol > methanol > n-butanol > ethyl acetate. Acetone was the best solvent for extracting polyphenols from barley bran among the other solvents evaluated. The polyphenol extraction amount is related to the content and composition of phenolics in raw materials, solvent polarity, and cell permeability. However, solvents primarily affect the extraction efficacy for the same raw materials [25]. Solvents such as acetone, ethanol, methanol, n-butanol, and ethyl acetate are frequently used in plant extract preparation, with acetone extracts generally having the highest polyphenol content, consistent with our findings [26,27]. Furthermore, the total phenolic content of the different solvent extracts evaluated was 23.08–78.54-fold higher than the total flavonoid content, indicating a low concentration of flavonoids in highland barley bran polyphenols. This was in agreement with Dang et al.’s findings on whole-grain testing of highland barley with different grain colors [5].

### 2.2. Composition Analysis of Phenolic Compounds

Forty-five polyphenol compounds were detected in the different solvent extracts of highland barley (Table 2), with the most abundant being diosmetin (0.00–185.39 μg/g), luteolin (0.00–39.81 μg/g), protocatechuic acid (14.16–16.93 μg/g), vanillic acid (7.50–13.17 μg/g), ferulic acid (6.12–20.98 μg/g), protocatechuic (0.05–12.17 μg/g), diosmin (0.91–12.79 μg/g), isoquercin (1.88–9.68 μg/g), catechin (6.83–10.40 μg/g) and isovitexin (6.99–10.05 μg/g). Diosmetin and luteolin were found in very low concentrations in methanol extracts, the same as phlorogucinol in n-butanol extracts. Catechin, *p*-hydroxybenzoic acid, caffeic acid, syringic acid, vanillin, *p*-coumaric acid, homoorientin, and quercetin were found at intermediate concentration levels in the different extracts evaluated. Other phenolic compounds, such as 2,4-dihydroxybenzoic acid, pyrogallol, 6-gingerol, hesperidin, myricetin, phloretin, kaempferol, tectorigenin and psoralidin, were commonly found at low levels. According to previous reports, bran significantly contributes to polyphenol enrichment in barley and highland barley. The main polyphenols found in barley bran include ferulic acid, protocatechuic acid, chlorogenic acid, benzogenic acid, catechin, kaempferol, vanillic acid, *p*-coumaric acid, and others. We successfully identified all these compounds in our study in different solvent extracts of highland barley bran [5,28,29]. López-Perea et al. recovered gallic acid, chlorogenic acid, *p*-coumaric acid, ferulic acid, benzoic acid, quercetin, and kaempferol in an 80% methanol extract, 80% ethanol extract, and 50% acetone extract of barley, similarly to our study [30]. However, their concentrations differed in the present work, which could be attributed to differences in background values and the form of polyphenol compounds in different raw materials, as well as the polyphenol compounds dissolution properties in different solvent systems [9].

Overall, four kinds of polyphenols including veratric acid, procyanidin B2, vanillin, and isoquercitrin in the methanol extract contained a far greater content than the other solvents. A higher concentration of *o*-coumaric acid was recovered in the ethanol extract than in the other solvents. Acetone extracts had significantly higher epicatechin content than other solvents. Diosmetin, luteolin, protocatechuic acid, catechin, isovitexin, quercetin, homoorientin, homogentisic acid, *p*-coumaric acid, maltol, gallic acid, naringenin, myricetin, kaempferol, and tectorigenin concentration was significantly higher in the n-butanol extracts than in other solvents. Seven polyphenols were detected, including *p*-hydroxybenzoic acid, vanillic acid, caffeic acid, syringic acid, ferulic acid, benzoic acid, and salicylic acid in the ethyl acetate extracts at significantly higher concentrations than in other solvents. These findings revealed that the different polar organic solvents significantly affected the enrichment of highland barley bran polyphenols in the extract, with n-butanol having a higher enrichment potential for flavonoids and ethyl acetate having a higher enrichment potential for phenolic acids. Compared to n-butanol and ethyl acetate extracts, methanol, ethanol, and acetone extracts were rich both in flavonoids and phenolic acids. Teber et al. found that the contents of *p*-coumaric acid, trans-ferulic acid and syringic in acid and alkaline extracts of barley husks were all high, but the contents of quercetin-3-o-galactoside, naringenin, silymarin and epicatechin, etc., were low. There were obvious differences in the types and contents of polyphenols in the two extraction solvents. The phenolics composition and content in the above research was inconsistent with the results of this study, further indicating that both solvents and raw materials have an important impact on the enrichment of polyphenols [31]. Furthermore, the total phenolic content of the five extracts was as follows: n-butanol (323.44 μg/g) > ethanol (256.55 μg/g) > acetone (209.24 μg/g) > ethyl acetate (202.50 μg/g) > methanol (104.31 μg/g). This result contradicts the chemical method determination results of total phenol content. On the one hand, total phenols and total flavonoids determined by chemical approaches are measured in gallic acid and rutin equivalents, respectively, whereas total phenols determined by liquid chromatography are quantified in comparison to specific polyphenol standards as controls. On the other hand, highland barley bran is rich in phenolic compounds, and there are other unknown phenolic compounds to be identified [5,32]. In conclusion, the composition and content of polyphenol compounds enriched in the bran of highland barley were different in the solvent systems used. Appropriate solvents can be chosen based on the research purpose and the downstream applications.

### 2.3. Antioxidant Capacity of the Extracts

**Figure 1:**
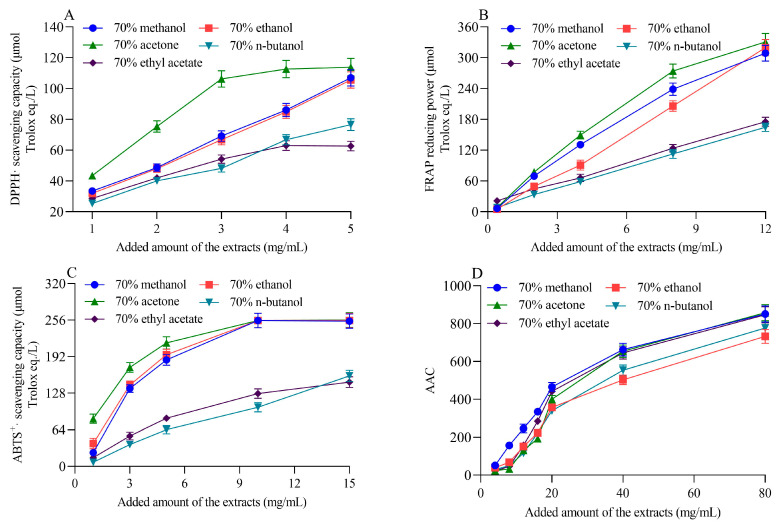
DPPH· radical scavenging capacity (**A**), FRAP reducing power (**B**), ABTS^+^· radical scavenging capacity (**C**) and antioxidant capacity in β-carotene-linoleic acid system (**D**) of different solvent extracts of black highland barley bran.

The DPPH· radical scavenging ability, FRAP reducing power, and ABTS^+^· radical scavenging ability of different solvent extracts of highland barley bran increased with an increasing extract dosage, showing a dose-dependent relationship (Figure 1). This phenomenon has been commonly observed in the antioxidant activity studies of different plant extracts [33,34]. The antioxidant capacity of the barley bran acetone extract was overall the highest among all extracts (Figure 1A–C). It was followed by the methanol and ethanol extracts, with the former having a slightly higher overall antioxidant capacity than the latter. The antioxidant capacities of the n-butanol and ethyl acetate extracts were comparable, and both were significantly lower than the other extracts across the concentration range evaluated. The methanol extract was slightly more resistant to bleaching in the β-carotene-linoleic acid antioxidant system than other solvents when the extract added was less than 40 mg/mL (Figure 1D). However, when the extract added was greater than 40 mg/mL, no significant differences (*p* > 0.05) were observed between the methanol, ethyl acetate, and acetone extracts. In the β-carotene-linoleic acid antioxidant system, the n-butanol extract was slightly more resistant to bleaching compared to the ethanol extract. Still, both were significantly lower than the other three extracts (*p* < 0.05). Taken together, the acetone extract of highland barley bran exhibited the highest antioxidant capacity, followed by the methanol and ethanol extracts. In contrast, n-butanol and ethyl acetate extracts had relatively low antioxidant capacity. These results were consistent with the results of total phenol content determined by chemical methods (Table 1). Liu et al. evaluated the antioxidant activity of 70% acetone extract, 70% ethanol extract and 70% methanol extract of barley and showed that the 70% acetone extract had the highest content of total phenolics and proanthocyanidins and the strongest antioxidant activity, followed by 70% ethanol extract and 70% methanol extract, which was in agreement with the results of this study [35]. Bulut et al. extracted phenolics from thermo-tolerant *Scenedesmus* sp. (Chlorophyta) with ethanol–water (3:1, *v*/*v*) and ethyl acetate and observed that ethanol–water (3:1, *v*/*v*) extracts exhibited significantly higher DPPH· radical scavenging and FRAP reducing ability compared to ethyl acetate extracts [36]. In acetone and ethyl acetate extracts from *Foeniculum vulgare* leaves, the DPPH· radical scavenging rate of the acetone extract was higher compared to the ethyl acetate extract [34]. These findings suggest that 70% acetone was an effective solvent for antioxidant compound enrichment in highland barley bran.

### 2.4. Enzyme Inhibitory Activity of the Extracts

**Figure 2:**
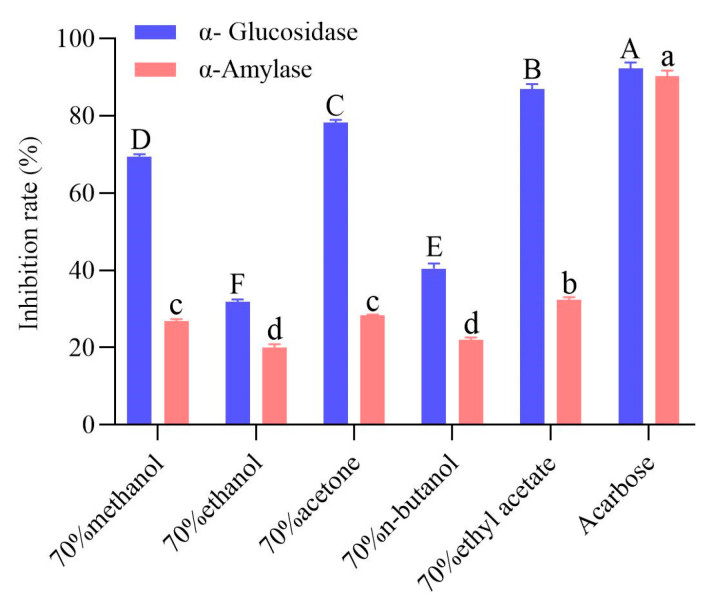
α-Glucosidase and α-amylase inhibition rates of different solvent extracts of black highland barley bran. The uppercase and lowercase letters in the figure respectively indicate significant differences in the inhibition rates of the extracts for α-glucosidase and α-amylase (*p* < 0.05).

The different solvent extracts of highland barley bran inhibited both α-glucosidase and α-amylase, with significant differences in inhibition rates (Figure 2). Thus, barley bran could be a promising bioactive substance source that regulates glucose metabolism. The α-glucosidase and α-amylase inhibitory activity of the extracts were 34.45–94.32% and 22.08–35.92%, respectively, of that of acarbose. Among the extracts, the ethyl acetate extract inhibited α-glucosidase and α-amylase by 86.95% and 32.41%, respectively, significantly higher than the other extracts (*p* < 0.05). The acetone extract exhibited α-glucosidase and α-amylase inhibitory activity secondly only to ethyl acetate extract, followed by the methanol extract, and the n-butanol and ethanol extracts were lower. This result showed that different solvent extracts of highland barley bran had good potential to inhibit α-glucosidase and α-amylase, corresponding to the findings of Deng et al. who confirmed that the polyphenol-rich extract of highland barley varieties had favorable hypoglycemic activity [23]. Active compounds from plant extracts are shown to be potent α-glucosidase and α-amylase inhibitors [10,37]. Polyphenol compounds have an inhibitory activity in plant extracts [12,38]. The inhibition rate of α-glucosidase from bound phenol was greater than that of free phenol in highland barley with different grain colors. Moreover, differences were observed between different highland barley varieties, with the polyphenol extract from purple highland barley and the anthocyanin extract from the black highland barley having the strongest α-glucosidase inhibitory activity. Furthermore, a strong relationship was observed between specific polyphenol compounds and α-glucosidase activity [12]. Among various phenolic compounds, ferulic acid and catechin have been proven to possess obvious inhibitory activity toward α-glucosidase and α-amylase via specific action sites, such as Arg^407^, Asp^326^, Arg^197^ residues, etc., in α-glucosidase and Asp^197^, Glu^233^, Asp^300^ residues, etc., in α-amylase [39]. In the present work, we found that the ethyl acetate extract with the strongest inhibitory activity also contained the richest ferulic acid, which was consistent with the existing reports. Kocak et al. measured the in vitro enzyme inhibitory activity of *Stachys annua* subsp. *Annua var. annua* extracts in methanol, ethyl acetate, and aqueous extracts. They discovered that the ethyl acetate extract exhibited the strongest inhibitory activity against acetylcholinesterase, butyrylcholinesterase, tyrosinase, α-glucosidase, and α-amylase, linked to its high total flavonoids, saponins, (+)-catechin, and *p*-hydroxybenzoic acid levels [40]. The in vitro enzyme inhibitory activity of *Symphytum anatolicum* ethyl acetate, methanol, and water extracts was investigated, with the methanol extract having the strongest tyrosinase inhibitory activity. In contrast, ethyl acetate extract had the strongest α-amylase inhibitory activity. Notably, the caffeic acid and verbascoside concentrations were found to be highly correlated with tyrosinase and α-amylase inhibitory activity [9]. The enzyme inhibitory activity of barley bran extract should be related to polyphenol compounds in the extract. In contrast, the difference in enzyme inhibitory activity of different extracts is potentially a result of the differences in solubility in the different solvents of the compounds exerting inhibitory enzyme effects [9].

### 2.5. Correlations between Antioxidant Capacity, Enzymatic Inhibitory, and Phenolics

A strong correlation was observed between the extracts’ total phenolic and total flavonoid contents and their antioxidant capacity of DPPH, ABTS, and FRAP, with correlation coefficients ranging from 0.706 to 0.971 (Table 3). The correlation between the total phenolic content and the antioxidant capacity of DPPH, ABTS, and FRAP was statistically significant (*p* < 0.05). This implied that the antioxidant activity of different highland barley bran solvent extracts was closely related to their phenolic content. This was consistent with the consensus of polyphenol antioxidant capacity in plants [9,27]. Positive correlations were observed between 25 monomeric polyphenol compounds and the extracts’ antioxidant and enzyme inhibitory activities. The correlation coefficient between the three monomeric phenol concentrations and the DPPH· radical scavenging ability exceeded 0.7, with epicatechin having the highest, statistically significant correlation (r = 0.963, *p* < 0.05). The correlation coefficients between nine monomeric phenol concentrations and ABTS^+^· radical scavenging ability exceeded 0.7. The correlation coefficients of chlorogenic acid and kaempferol-3-o-rutinoside concentrations and ABTS^+^· radical scavenging ability reached 0.976 and 0.902, respectively (*p* < 0.05). The correlation coefficients between nine monomeric phenols and FRAP reducing ability was greater than 0.7, while chlorogenic acid, kaempferol-3-o-rutinoside and 2-hydroxyphenylacetic acid concentrations, and FRAP reducing ability reached a 0.984, 0.962 and 0.931 correlation, respectively (*p* < 0.05). *o*-coumaric acid, 2,4-dihydroxybenzoic acid, and vanillin all exhibited a greater than 0.6 correlation with AAC. Thus, polyphenol compounds such as epicatechin, chlorogenic acid, kaempferol-3-o-rutinoside, 2-hydroxyphenylacetic acid, and *o*-coumaric acid might mainly contribute to the extract’s overall antioxidant capacity. Furthermore, different monomeric polyphenol compounds showed certain selectivity for different antioxidant systems [1]. Among the polyphenols from highland barley with different grain colors, protocatechuic acid and catechin might be the main contributors to DPPH· free radical scavenging ability. On the other hand, chlorogenic acid and catechin might be the main contributors to FRAP reducing ability, while benzoic acid might be the main contributor to ABTS^+^· free radical scavenging ability [5]. Yang et al. discovered that in blue-colored highland barley, 2,4-dihydroxybenzoic acid and protocatechuic acid were the main contributors to the free phenol extract scavenging capacity against DPPH· and ABTS^+^· free radicals, while chlorogenic acid, vanillic acid, ferulic acid, and quercetin contributed to the bound phenol extract free radicals scavenging capacity [1]. The main contributors to DPPH· and ABTS^+^· radical scavenging ability and FRAP reducing ability in Tunisian barley phenolic extracts were catechin-3-glucose and ferulic acid [41]. These results differed from those found in this paper, which could result from different varieties studied, growth environments, different parts of the raw materials used, and different phenolic enrichment processes [28].

Among the 25 monomeric phenols identified, the correlation between the contents of the eight polyphenols and the α-glucosidase and α-amylase inhibition rate of the extracts was greater than 0.5. Among those were *p*-hydroxybenzoic acid, vanillic acid, 2,4-dihydroxybenzoic acid, caffeic acid, syringic acid, ferulic acid, and salicylic acid, indicating that these monomeric phenols might contribute to the blood glucose regulatory properties of the different solvent extracts of highland barley bran. The correlation coefficients between benzoic acid content and α-glucosidase and α-amylase inhibition rate were 0.730 and 0.828, respectively, indicating that benzoic acid might be the main contributor to the extract’s enzyme inhibitory ability. Jin et al. discovered compounds in polyphenol extracts of colored-grain highland barley, such as *o*-coumaric acid, vanillic acid, pelargonidin-3-glucoside, and petunidin, which were closely correlated to α-glucosidase inhibitory activity [12]. Verbascoside has been shown to be a major contributor to α-amylase inhibitory activity in *Symphytum anatolicum* solvent extracts [9]. These findings differed from this study, demonstrating that differences in extraction solvents and raw materials significantly impact the enzyme inhibitory activity of polyphenol compounds [40]. Furthermore, there was a significant positive correlation between the extract’s α-glucosidase inhibitory activity and AAC, with a correlation coefficient of 0.934 (*p* < 0.05), indicating that the AAC could be used as a proxy for the extract’s α-glucosidase inhibitory ability. Effectively, it was difficult for a monomer polyphenol to reflect the in vitro biological activity of the extracts. The suppression effect of different solvent extracts of highland barley bran might be the synergistic and additive effects of phenolics [23]. In conclusion, highland barley bran phenolic compounds extracted with different solvents have a high correlation with antioxidant capacity, α-glucosidase inhibitory activity, and α-amylase inhibitory activity. Thus, highland barley bran is an excellent functional food raw material for active antioxidant ingredients and enzyme inhibitors.

## 3. Materials and Methods

### 3.1. Chemicals and Materials

The variety of 947 black highland barley was cultivated by Qinghai Academy of Agriculture and Forestry Sciences and was planted in 2021 in the experimental field (Xining, 36°67′ N 101°77′ E, altitude 2300 m). The black highland barley bran was collected by Qinghai New Lilac Cereals and Oils Co., Ltd. after 10 passes of milling and peeling, with a particle size of 100 meshes. The β-carotene, linoleic acid, 1,1-diphenyl-2-picrylhydrazylradical (DPPH), 2,4,6-tripyridyl-s-triazine (TPTZ), 2,20-azinobis-(3-ethylbenzthiazoline-6-sulfonate) (ABTS), 6-hydroxy-2,5,7,8-tetramethylchroman-2-carboxylic acid (Trolox), and *p*-nitrophenyl-α-glucopyranoside (pNPG) with BR level were provided by Sigma Co. (STL, MO, USA). The 48 kinds of polyphenol standards including 26 kinds of phenolic acids (phlorogucinol, gallic acid, pyrogallol, homogentisic acid, protocatechuic acid, procyanidin B2, 4-hydroxybenzoic acid, chlorgenic acid, vanillic acid, 4-hydrosxybenzaldehyde, 2-hydroxyphenylacetic acid, 2,4-dihydroxybenzoic acid, caffeic acid, syringic acid, vanillin, procyanidin A2, maltol, trans-4-hydroxycinamic acid, sesamol, ferulic acid, 3,4-dimethoxybenzoic, benzoic acid, salicylic acid, 2-hydroxycinnamic acid, 6-gingerol, taxifolin) and 22 kinds of flavonoids (catechin, cianidanol, epicatechin, puerarin, vitexin, naringin, hesperidin, homoorientin, isovitexin, rutin, isoquercitrin, diosmin, kaempferol-3-o-rutinoside, myricetin, naringenin, phloretin, quercetin, tectorigenin, luteolin, kaempferol, diosmetin, psoralidin) with purity ≥ 98%, and α-amylase from porcine pancreas (10 U/mg) and α-glucosidase from yeast (50 U/mg) were purchased from Shanghai Yuanye Bio-Technology Co., Ltd. (Shanghai, China). The Folin–Ciocalteu reagent with GR level, acarbose with purity ≥95% and 3,5-dinitrosalicylic acid (DNS) reagents were purchased by Beijing Solarbio Science & Technology Co., Ltd. (Beijing, China). Deionized water was used throughout the test. Glacial acetic acid and methanol with chromatographic grade were used for phenolic composition analysis by HPLC-MS/MS. All the other chemicals and reagents used in the experiments were domestic analytical pure grade.

### 3.2. Preparation of Extracts of Black Highland Barley Bran

First, 2 g of black highland barley bran was mixed with 70% methanol, 70% methanol, 70% ethanol, 70% acetone, 70% n-butanol or 70% ethyl acetate at a material-to-liquid ratio of 1:20 (g/mL). The mixture was extracted by shaking in a water bath oscillator at 25 °C for 12 h, and then, the supernatant was collected while the obtained residue was extracted again with the same procedure. The supernatants were merged and evaporated to dryness at 45 °C under vacuum, and the final residue was resolved with 10 mL methanol and subsequently filtrated using 0.45 μm organic membrane to obtain the extract. All the extracts were stored in the dark at −20 °C until use.

### 3.3. Assay of Total Phenolic Content

Folin–Ciocalteu method was used to detect the total phenolic content of the extracts [42]. Specifically, 500 μL of deionized water and 125 μL of Folin–Ciocalteu reagent were added to 125 μL of the extract in turn, and then, the mixture was left to react at room temperature for 6 min. Next, 1.25 mL of 7% Na_2_CO_3_ solution was added, and the total volume of the mixture was replenished with water to 3 mL. The obtained sample was shielded from light at room temperature for 1.5 h. Finally, the sample absorbance was collected at 760 nm via a spectrometer (N4S, Yidian, Shanghai, China), and total phenolic content was calculated using gallic acid as the standard (mg/100 g DW).

### 3.4. Assay of Total Flavonoids Content

The colorimetric method was used to detect the total flavonoids content of the extracts [42]. Specifically, 200 μL of 5% NaNO_2_ solution was mixed with 1 mL of the extract for 6 min, and then, 200 μL of 10% AlCl_3_·6H_2_O solution was added to the above mixture for another 6 min. Next, after adding 2 mL of 4% NaOH solution to the mixture and reacting at room temperature in the dark for 15 min, the absorbance of the sample was collected at 510 nm. Total flavonoids content in the extract was calculated using catechin as the standard (mg/100 g DW).

### 3.5. Composition Analysis of Phenolics in Extracts

The phenolic compounds of extracts of different solvents were detected by HPLC-MS/MS (Q-Exactive, Dionex Ultimate 3000 RSLC, ThermoFisher, Waltham, MA, USA) using a Hypersil GOLD aQ column (100 × 2.1 mm) and mass detector. The mobile phase A was distilled water with 0.9% glacial acetic acid, and the mobile phase B was methanol. The column temperature was 30 °C, the injection volume of the sample was 1 μL, and the flow rate was 0.3 mL/min. The elution program was as follows: 0–9 min, 20–100%B; 9–10 min, 100%B; 10–11 min, 100~20%B; 11–14 min, 20%B. The mass spectrometry conditions were as follows: The electrospray ion source (ESI) was used with a spray voltage of 2.8 kV. The positive/negative ion scanning mode was adopted in a full MS scan range of 100–850 m/z. The sheath gas (N_2_) and assist gas (N_2_) flows were 40 and 10 units/min, respectively. The capillary temperature and heater temperature were both 300 °C.

### 3.6. Determination of DPPH· Free Radical Scavenging Capacity

A method described by Abu Bakar et al. with some modifications was adopted to determine the DPPH· free radical scavenging capacity of the extracts [43]. In detail, 4.5 mL of 0.1 mmol/L DPPH–methanol solution was added to 1 mL of the extract, and the mixture was kept in darkness for 30 min. After the reaction, the absorbance of the sample was collected at 517 nm, and the blank zero was set with methanol instead of the extract. DPPH· radical scavenging capacity of the extract was calculated using Trolox as the standard (µmol Trolox eq./100 g DW).

### 3.7. Determination of ABTS^+^· Free Radical Scavenging Capacity

ABTS^+^· radical scavenging capacity of the extract was detected based on the method by Guo et al. with some modifications [44]. Firstly, the ABTS^+^· working solution was prepared by adding 88 μL of 140 mmol/L potassium persulfate solution into 5 mL of 7 mmol/L ABTS solution, and the mixed solution was then kept in the dark for 12–16 h. The above stock solution was diluted to an appropriate absorbance (0.7 ± 0.02) using methanol at a volume ratio of 1:100 before use. To a test tube, 200 μL of the extract and 4 mL of diluted ABTS^+^· working solution was thoroughly mixed and kept in the dark for 30 min. Finally, the absorbance of the sample was recorded at 734 nm, and the blank zero was set with methanol instead of the extract. ABTS^+^· radical scavenging capacity of the extract was calculated using Trolox as the standard (µmol Trolox eq./100 g DW).

### 3.8. Determination of Ferric Reducing Antioxidant Power

Ferric reducing antioxidant power (FRAP) of the extract was detected based on the method by Benzie et al. with some modifications [45]. The FRAP working solution was a mixture of 300 mmol/L of sodium acetate buffer at pH 3.6, 10 mmol/L of TPTZ solution and 20 mmol/L of FeCl_3_ solution (10:1:1, *v*/*v*/*v*). This work solution was ready-made and preheated at 37 °C prior to use. Typically, 4.5 mL of FRAP working solution was added to 1 mL of the extract, and the mixture was thoroughly mixed and allowed to react in darkness for 30 min. Then, the absorbance of the sample was recorded at 593 nm, and the blank zero was set with methanol instead of the extract. FRAP reducing antioxidant power of the extract was calculated using Trolox as the standard (µmol Trolox eq./100 g DW).

### 3.9. Assay of Antioxidant Capacity in β-Carotene-Linoleic Acid System

The antioxidant capacity of the extract in β-carotene-linoleic acid antioxidant system was determined according to the method by Li and Zhou with some modifications [46]. Firstly, 2 mL of β-carotene solution, 45 mg of linoleic acid and 350 mg of Tween-40 were mixed thoroughly, and then, the chloroform in mixture was removed by rotary evaporation. The residue was further filled with distilled water to a total volume of 100 mL to obtain the β-carotene-linoleic acid emulsion. Typically, 100 μL of the extract was added to 4 mL of β-carotene-linoleic acid emulsion, and then, the mixture was placed in a water bath at 50 °C to allow for thermal oxidation for 60 min. Then, the absorbance of the sample before and after hot oxidation was measured at 470 nm. In the control group, methanol was used instead of the extract, and the absorbance values of the solution before and after thermal oxidation were also measured. The emulsion without β-carotene was used as blank to correct the background absorption. Antioxidant activity coefficient (AAC) was calculated by the following formula:

AAC = [(As_60_ − Ac_60_)/(Ac_0_ − Ac_60_)] × 1000 (1)

where As_60_ is the absorption value of the sample after thermal oxidation for 60 min; Ac_60_ is the absorption value of the control after thermal oxidation for 60 min; Ac_0_ is the absorption value of the control before thermal oxidation.

### 3.10. Inhibitory Activity of α-Amylase

The α-amylase inhibitory activity of the extract was determined according to the method described by Tian et al. with some modification and the acarbose was used as positive control [47]. Firstly, 500 μL of the extract and equal volume of α-amylase solution (2.0 U/mL) prepared using 0.02 mol/L phosphate buffer (pH = 6.9) were thoroughly mixed, and then, the mixture was incubated at 37 °C for 10 min. The above mixture was incubated at 37 °C for another 10 min after adding 500 μL of 1% soluble starch aqueous solution. Subsequently, 1 mL of DNS reagent as color indicator was added to stop the reaction, and then, the mixture was treated using boiling water for 5 min. The mixture was further diluted by adding 10 mL of distilled water to a total volume of 12.5 mL. The absorbance of the obtained sample was measured at 540 nm, and the α-amylase inhibition rate was calculated according to Formula (2).

Inhibitory rate (%) = [1 − (A_1_ − A_2_)/A_3_] × 100 (2)

where A_1_ is the absorbance value of the sample; A_2_ is the background absorbance value of the phosphate buffer replacing the α-amylase solution in the reaction system; A_3_ is the blank control absorbance value of the phosphate buffer replacing the extract solution in the reaction system.

### 3.11. Inhibitory Activity of α-Glucosidase

The α-glucosidase inhibitory activity of the extract was determined according to the method described by Jin et al. with some modification, and the acarbose was used as positive control [12]. To a 96-well plate, 40 μL of the extract and 30 μL of α-glucosidase solution (0.2 U/mL) prepared using 0.1 mol/L phosphate buffer (pH = 6.8) were added, and then, the mixture was mixed well and incubated at 37 °C for 10 min. Next, 30 μL of 5 mmol/L pNPG (dissolved in 0.1 mol/L phosphate buffer at pH 6.8) was added to the wall, and the mixture was incubated at 37 °C for 30 min. Finally, 100 μL of 1 mol/L Na_2_CO_3_ aqueous solution was added to the mixture to terminate the reaction. The total volume of the sample was 200 μL. The absorbance of the obtained sample was measured at 405 nm, and the α-glucosidase inhibition rate was calculated according to Formula (3).

Inhibitory rate (%) = [1 − (B_1_ − B_2_)/B_3_] × 100 (3)

where B_1_ is the absorbance value of the sample; B_2_ is the background absorbance value of the phosphate buffer replacing the glucosidase solution in the reaction system; B_3_ is the blank control absorbance value of the phosphate buffer replacing the extract in the reaction system.

### 3.12. Statistical Analysis

The data were measured in triplicate and reported as mean ± SD (standard deviation). The data were analyzed using Excel 2007 (Microsoft, Redmond, WA, USA), and related images were drawn using GraphPad Prism 8 (GraphPad, SD, CA, USA). Significant differences among the means were calculated using a Student–Newman–Keuls q (SNK-q) test, and statistical significance was defined as *p* < 0.05. The correlation analysis was performed using the Pearson two-sided test through Origin 2019 (OriginLab, Northampton, MA, USA).

## 4. Conclusions

Different solvent extracts of highland barley bran were evaluated in this study. They resulted in different polyphenol content and composition, antioxidant activity, and enzyme inhibitory activity of the extracts in vitro. The highest polyphenol content and antioxidant capacity were observed in the 70% acetone extract, followed by the 70% methanol extract and 70% ethanol extract, while 70% n-butanol extract and 70% ethyl acetate extract showed lower levels; 70% acetone was an effective solvent for antioxidant compound enrichment in highland barley bran. The major polyphenol compounds identified in the different extracts were diosmetin, luteolin, protocatechuic acid, vanillic acid, ferulic acid, protocatechuic, diosmin, isoquercitrin, catechin and isovitexin, and their content distributions varied depending on the extraction solvent. The 70% ethyl acetate extract exhibited the highest inhibition of the α-glucosidase and α-amylase enzymes, followed by the 70% acetone extract and 70% methanol extract, while 70% n-butanol and 70% ethanol extracts exhibited lower measured values. The inhibitory activity of different solvent extracts of highland barley bran on α-glucosidase and α-amylase could respectively reach to 34.45–94.32% and 22.08–35.92% of that of acarbose, exhibiting a good hypoglycemic potential. A high correlation was observed between phenolic compounds and the antioxidant capacity, α-glucosidase inhibitory activity, and α-amylase inhibitory activity of different solvent extracts. Highland barley bran is an excellent functional food raw material for the evaluation and functional implementation of antioxidant active ingredients and enzyme inhibitors associated with glucose metabolism. The findings can serve as the theoretical foundation for further research into highland barley bran functional properties and their applications in health-promoting food products.

## Figures and Tables

**Table 1:** Phenolic content of different solvent extracts of black highland barley bran.

Phenolic Content (mg/100 g DW)	70% Methanol	70% Ethanol	70% Acetone	70% n-Butanol	70% Ethyl Acetate
TPC	253.48 ± 9.54 ^b^	256.86 ± 6.98 ^b^	281.98 ± 4.29 ^a^	206.35 ± 6.02 ^c^	197.93 ± 5.69 ^c^
TFC	9.85 ± 0.78 ^b^	10.00 ± 0.82 ^b^	12.22 ± 0.43 ^a^	8.00 ± 0.65 ^c^	2.52 ± 0.24 ^d^

Note: Different letters after data within the same line indicate significant differences (*p* < 0.05). TPC and TFC represent total phenolic content and total flavonoids content detected by chemical method, respectively.

**Table 2:** Profile of phenolic compounds in different solvent extracts of black highland barley bran.

Phenolic Compounds (μg/g)	70% Methanol	70% Ethanol	70% Acetone	70% n-Butanol	70% Ethyl Acetate
Gallic acid	0.29 ± 0.01 ^c^	0.25 ± 0.01 ^d^	0.34 ± 0.01 ^b^	0.54 ± 0.04 ^a^	0.15 ± 0.01 ^e^
Homogentisic acid	0.02 ± 0.00 ^c^	2.02 ± 0.51 ^a^	1.74 ± 0.17 ^b^	2.03 ± 0.04 ^a^	0.01 ± 0.01 ^d^
Protocatechuic acid	14.62 ± 0.33 ^c^	14.16 ± 0.23 ^c^	15.98 ± 0.25 ^ab^	16.93 ± 0.69 ^a^	15.34 ± 0.38 ^b^
*p*-Hydroxybenzoic acid	2.79 ± 0.06 ^b^	2.65 ± 0.04 ^bc^	2.53 ± 0.03 ^c^	2.5 ± 0.23 ^c^	3.62 ± 0.04 ^a^
Chlorgenic acid	0.22 ± 0.01 ^a^	0.19 ± 0.01 ^a^	0.23 ± 0.10 ^a^	0.02 ± 0.01 ^b^	0.02 ± 0.01 ^b^
Vanillic acid	10.7 ± 0.09 ^b^	10.06 ± 0.27 ^b^	9.13 ± 0.55 ^c^	7.5 ± 1.15 ^d^	13.17 ± 0.10 ^a^
2-Hydroxyphenylacetic acid	0.16 ± 0.06 ^a^	0.15 ± 0.04 ^a^	0.14 ± 0.03 ^b^	0.07 ± 0.02 ^d^	0.1 ± 0.01 ^c^
2,4-Dihydroxybenzoic acid	0.00 ± 0.00 ^a^	0.00 ± 0.00 ^a^	0.00 ± 0.00 ^a^	0.00 ± 0.00 ^a^	0.00 ± 0.00 ^a^
Caffeic acid	1.95 ± 0.08 ^b^	1.92 ± 0.05 ^b^	1.98 ± 0.09 ^b^	2.84 ± 0.17 ^b^	6.16 ± 0.07 ^a^
Syringic acid	2.89 ± 0.10 ^b^	2.55 ± 0.02 ^c^	2.43 ± 0.04 ^c^	2.12 ± 0.28 ^d^	3.13 ± 0.04 ^a^
*p*-Coumaric acid	1.15 ± 0.03 ^b^	1.19 ± 0.02 ^b^	1.02 ± 0.01 ^c^	1.87 ± 0.01 ^a^	1.2 ± 0.00 ^b^
*o*-Coumaric acid	0.03 ± 0.01 ^c^	0.09 ± 0.05 ^a^	0.05 ± 0.01 ^b^	0.01 ± 0.00 ^e^	0.02 ± 0.01 ^d^
Ferulic acid	7.07 ± 0.15 b	6.55 ± 0.06 c	6.1 ± 0.14 c	7.06 ± 0.61 b	20.98 ± 0.24 a
Veratric acid	0.41 ± 0.04 ^a^	0.07 ± 0.00 ^d^	0.11 ± 0.02 ^b^	0.09 ± 0.01 ^c^	0.11 ± 0.01 ^b^
Benzoic acid	0.96 ± 0.24 ^b^	0.78 ± 0.13 ^d^	0.82 ± 0.04 ^c^	0.67 ± 0.09 ^e^	1.47 ± 0.21 ^a^
Salicylic acid	0.08 ± 0.01 ^b^	0.07 ± 0.00 ^c^	0.08 ± 0.02 ^b^	0.07 ± 0.01 ^c^	0.13 ± 0.01 ^a^
Procyanidin B2	1.43 ± 0.02 ^a^	0.02 ± 0.01 ^d^	0.01 ± 0.00 ^e^	0.07 ± 0.02 ^c^	0.31 ± 0.01 ^b^
Phlorogucinol	10.7 ± 0.24 ^b^	12.00 ± 1.52 ^a^	12.17 ± 1.14 ^a^	0.05 ± 0.00 ^c^	12.71 ± 0.95 ^a^
Pyrogallol	0.00 ± 0.00 ^a^	0.01 ± 0.00 ^a^	0.01 ± 0.00 ^a^	0.01 ± 0.00 ^a^	0.01 ± 0.00 ^a^
Maltol	0.79 ± 0.23 ^b^	0.58 ± 0.18 ^d^	0.46 ± 0.11 ^e^	0.88 ± 0.23 ^a^	0.7 ± 0.05 ^c^
Sesamol	0.01 ± 0.00 ^a^	0.01 ± 0.01 ^a^	0.01 ± 0.00 ^a^	0.01 ± 0.00 ^a^	0 ± 0.00 ^b^
6-Gingerol	0.04 ± 0.02 ^a^	0.04 ± 0.02 ^a^	0.03 ± 0.00 ^b^	0.04 ± 0.02 ^a^	0.03 ± 0.01 ^b^
Taxifolin	0.26 ± 0.00 ^c^	0.3 ± 0.00 ^b^	0.27 ± 0.00 ^c^	0.45 ± 0.02 ^a^	0.47 ± 0.01 ^a^
4-Hydrosxybenzaldehyde	0.37 ± 0.02 ^a^	0.31 ± 0.01 ^c^	0.33 ± 0.02 ^bc^	0.35 ± 0.05 ^ab^	0.3 ± 0.01 ^c^
Vanillin	2.17 ± 0.05 ^a^	1.79 ± 0.05 ^c^	1.93 ± 0.17 ^b^	1.76 ± 0.16 ^c^	1.78 ± 0.09 ^c^
Catechin	8.81 ± 0.05 ^b^	9.23 ± 0.05 ^b^	9.01 ± 0.06 ^b^	10.40 ± 0.10 ^a^	6.83 ± 0.02 ^c^
Epicatechin	0.13 ± 0.01 ^b^	0.13 ± 0.00 ^b^	0.16 ± 0.01 ^a^	0.12 ± 0.01 ^bc^	0.11 ± 0.00 ^c^
Naringin	0.14 ± 0.01 ^a^	0.13 ± 0.00 ^a^	0.13 ± 0.01 ^a^	0.09 ± 0.01 ^b^	0.02 ± 0.00 ^c^
Hesperidin	0.06 ± 0.01 ^a^	0.05 ± 0.01 ^a^	0.05 ± 0.01 ^a^	0.04 ± 0.00 ^b^	0.05 ± 0.01 ^a^
Rutin	0.74 ± 0.02 ^a^	0.72 ± 0.01 ^a^	0.67 ± 0.03 ^b^	0.47 ± 0.02 ^c^	0.25 ± 0.00 ^d^
Myricetin	0.00 ± 0.00 ^c^	0.01 ± 0.00 ^b^	0.01 ± 0.00 ^b^	0.06 ± 0.01 ^a^	0.00 ± 0.00 ^c^
Naringenin	0.00 ± 0.00 ^e^	0.12 ± 0.01 ^c^	0.07 ± 0.01 ^d^	0.17 ± 0.01 ^a^	0.15 ± 0.00 ^b^
Phloretin	0.00 ± 0.00 ^a^	0.00 ± 0.00 ^a^	0.00 ± 0.00 ^a^	0.00 ± 0.00 ^a^	0.00 ± 0.00 ^a^
Quercetin	1.14 ± 0.02 ^d^	1.7 ± 0.05 ^b^	1.25 ± 0.01 ^c^	6.2 ± 0.18 ^a^	0.34 ± 0.02 ^e^
Isoquercitrin	9.68 ± 0.15 ^a^	8.51 ± 0.10 ^b^	6.99 ± 0.07 ^c^	5.56 ± 0.06 ^d^	1.88 ± 0.01 ^e^
Vitexin	0.18 ± 0.00 ^a^	0.18 ± 0.01 ^a^	0.19 ± 0.02 ^a^	0.16 ± 0.00 ^b^	0.11 ± 0.00 ^c^
Isovitexin	7.51 ± 0.05 ^c^	7.35 ± 0.05 ^c^	6.99 ± 0.08 ^d^	10.05 ± 0.22 ^a^	8.08 ± 0.20 ^b^
Kaempferol	0.04 ± 0.01 ^b^	0.03 ± 0.01 ^c^	0.01 ± 0.01 ^e^	0.05 ± 0.02 ^a^	0.02 ± 0.00 ^d^
Kaempferol-3-o-rutinoside	1.21 ± 0.02 ^a^	1.17 ± 0.04 ^a^	1.05 ± 0.03 ^b^	0.16 ± 0.01 ^d^	0.35 ± 0.21 ^c^
Diosmin	12.76 ± 0.25 ^a^	12.41 ± 0.30 ^a^	11.72 ± 0.24 ^b^	12.79 ± 1.33 ^a^	0.91 ± 0.01 ^c^
Homoorientin	2.79 ± 0.09 ^b^	2.98 ± 0.06 ^b^	3.04 ± 0.02 ^b^	3.93 ± 0.22 ^a^	1.77 ± 0.01 ^c^
Tectorigenin	0.01 ± 0.01 ^c^	0.02 ± 0.01 ^b^	0 ± 0.00 ^d^	0.05 ± 0.01 ^a^	0.02 ± 0.01 ^b^
Luteolin	0 ± 0.00 ^e^	22.68 ± 0.08 ^b^	14.82 ± 0.23 ^d^	39.81 ± 0.97 ^a^	16.59 ± 0.41 ^c^
Diosmetin	0 ± 0.00 ^e^	131.37 ± 0.31 ^b^	95.18 ± 0.32 ^c^	185.39 ± 1.95 ^a^	83.1 ± 1.78 ^d^
Psoralidin	0 ± 0.00 ^a^	0 ± 0.00 ^a^	0 ± 0.00 ^a^	0 ± 0.00 ^a^	nd
TPH	104.31 ± 5.21 ^d^	256.55 ± 8.77 ^b^	209.24 ± 5.53 ^c^	323.44 ± 12.49 ^a^	202.50 ± 4.98 ^c^

Note: Different letters after data within the same line indicate significant differences (*p* < 0.05); nd: not detected; TPH: total content of phenolic compounds detected by HPLC method.

**Table 3:** Pearson correlation coefficients between antioxidant capacity, enzyme inhibition activity and phenolic compounds.

Groups	DPPH	ABTS	FRAP	AAC	α-Glucosidase	α-Amylase
TPC	0.881 *	0.971 *	0.970 *			
TFC	0.706	0.805	0.781			
α-Glucosidase	0.346	0.186	0.028	0.934 *		
α-Amylase	0.191	0.011		0.869	0.982 *	
*p*-hydroxybenzoic acid				0.378	0.604	0.729
Chlorgenic acid	0.781	0.976 *	0.984 *	0.180	0.024	
Vanillic acid				0.417	0.608	0.684
2-Hydroxyphenylacetic acid	0.612	0.874	0.931 *	0.202	0.106	
2,4-Dihydroxybenzoic acid				0.642	0.693	0.746
Caffeic acid				0.259	0.515	0.668
Syringic acid		0.016	0.038	0.535	0.648	0.695
*o*-Coumaric Acid		0.376	0.514	0.716		
Ferulic acid				0.319	0.576	0.718
Veratric acid	0.054	0.386	0.280	0.569	0.300	0.211
Benzoic acid				0.525	0.730	0.828
Salicylic acid				0.457	0.679	0.798
Procyanidin B2		0.252	0.171	0.533	0.302	0.244
Phlorogucinol	0.502	0.555	0.626	0.368	0.506	0.469
Pyrogallol	0.805	0.483	0.433	0.064	0.141	0.054
Sesamol	0.173	0.403	0.322			
4-Hydrosxybenzaldehyde	0.018	0.238	0.089	0.227		
Vanillin	0.392	0.669	0.550	0.627	0.355	0.217
Epicatechin	0.963 *	0.866	0.807	0.187	0.102	
Naringin	0.574	0.791	0.784			
Hesperidin	0.413	0.717	0.744	0.409	0.313	0.215
Rutin	0.545	0.813	0.846			
Isoquercitrin	0.374	0.708	0.739			
Vitexin	0.623	0.798	0.798			
Kaempferol-3-o-rutinoside	0.638	0.902 *	0.962 *	0.093		
Diosmin	0.291	0.481	0.474			

Note: * indicates significance *p* < 0.05. TPC and TFC: total phenolic content and total flavonoids content detected by chemical method, respectively. AAC: antioxidant activity coefficient of the extracts in β-carotene-linoleic acid system.

## Data Availability

The data presented in this study are available within the article.

## Sample Availability

Samples of the compounds are not available from the authors.
